# From Non-Advanced Disease to Mast Cell Leukemia: Diagnostic, Morphologic, and Molecular Heterogeneity of Systemic Mastocytosis in a Single-Center Real-World Series

**DOI:** 10.3390/diagnostics16213499

**Published:** 2026-10-26

**Authors:** Alina Camelia Catana, Corina Porr, Lidia-Maria Mondoc, Liliana Mocanu, Ariela Olteanu, Livia Draghila, Gabriela Mariana Iancu

**Affiliations:** 1 Hematology Department, Faculty of Medicine, Lucian Blaga University of Sibiu, 550169 Sibiu, Romania; alinacamalia.catana@ulbsibiu.ro; 2 Clinic of Hematology, County Clinical Emergency Hospital Sibiu, Bulevardul Corneliu Coposu 2–4, 550245 Sibiu, Romania; 3 Allergology Department, Faculty of Medicine, Lucian Blaga University of Sibiu, 550169 Sibiu, Romania; 4 Allergology Department, County Clinical Emergency Hospital Sibiu, Bulevardul Corneliu Coposu 2–4, 550245 Sibiu, Romania; 5 Preclinical Department, Faculty of Medicine, Lucian Blaga University of Sibiu, 550169 Sibiu, Romania; 6 Ambulatory of Hematology, County Clinical Emergency Hospital Sibiu, Bulevardul Corneliu Coposu 2–4, 550245 Sibiu, Romania; 7 Department of Laboratory Medicine, County Clinical Emergency Hospital Sibiu, Bulevardul Corneliu Coposu 2–4, 550245 Sibiu, Romania; 8 Dermatology Department, Faculty of Medicine, Lucian Blaga University of Sibiu, 550169 Sibiu, Romania; gabriela.iancu@ulbsibiu.ro; 9 Clinic of Dermatology, County Clinical Emergency Hospital Sibiu, Bulevardul Corneliu Coposu 2–4, 550245 Sibiu, Romania

**Keywords:** systemic mastocytosis, mast cell leukemia, myeloid/lymphoid neoplasm with eosinophilia, diagnostic approach, serum tryptase, *KIT* D816V, *FIP1L1::PDGFRA*

## Abstract

**Background/Objectives:** Systemic mastocytosis (SM) is a heterogeneous clonal mast-cell neoplasm requiring integrated clinical, biochemical, morphological, immunophenotypic, and molecular assessment. This study aimed to characterize the diagnostic and biological heterogeneity of SM across different disease phenotypes. **Methods:** We retrospectively reviewed six patients with SM diagnosed and managed between 2018 and 2022 at a single center. Clinical presentations, organ involvement, serum tryptase concentrations, bone marrow morphology, immunophenotypic and molecular findings, treatment, and outcomes were analyzed. **Results:** The cohort included four males and two females aged 16–86 years. Cutaneous manifestations occurred in five patients and frequently preceded diagnosis. All patients had bone marrow involvement, with variable mast-cell infiltration and aberrant CD25 and/or CD2 expression. Serum tryptase concentrations ranged from 15.8 to 348 μg/L. The highest concentration occurred in Patient 5, who had a *FIP1L1::PDGFRA*-positive myeloid/lymphoid neoplasm with eosinophilia and prominent systemic mast-cell involvement. In contrast, Patient 6, diagnosed with mast cell leukemia, had a tryptase concentration of 28.5 μg/L despite extensive marrow and peripheral blood involvement. *KIT* mutations were detected in five patients, while Patient 6 tested negative. **Conclusions:** This series highlights the marked clinical and biological heterogeneity of SM and the potential discordance between serum tryptase concentrations, mast-cell burden, molecular findings, and disease aggressiveness. Accurate diagnosis and disease characterization require an integrated multidisciplinary approach.

## 1. Introduction

Systemic mastocytosis (SM) is a rare clonal hematologic neoplasm characterized by abnormal mast-cell proliferation and accumulation in extracutaneous organs, particularly the bone marrow. Other affected sites may include the gastrointestinal tract, liver, spleen, and skeletal system, while cutaneous involvement is also common [1,2,3,4,5]. Its clinical spectrum ranges from non-advanced disease, predominantly associated with mast-cell mediator-related symptoms, to advanced forms characterized by organ damage, hematologic complications, and reduced survival [1,2,3,4,5,6,7,8]. Activating mutations in *KIT*, particularly *KIT* D816V, are major molecular features of SM [3,4,5,6,7,8].

Clinical manifestations vary widely and often overlap with those of other hematologic, dermatologic, allergic, and gastrointestinal disorders. Cutaneous findings, including pruritus, flushing, and maculopapular or urticaria-like lesions, may precede systemic diagnosis [9,10]. Atypical cutaneous presentations, including unusual lesion morphology or distribution, may further complicate clinical recognition and delay diagnosis [11]. Gastrointestinal symptoms include abdominal pain, nausea, and diarrhea, whereas skeletal involvement may manifest as bone pain, osteoporosis, or osteolytic lesions [12,13,14,15,16]. Anaphylaxis is another important manifestation, particularly in patients with clonal mast-cell disease and Hymenoptera venom allergy [17,18,19,20,21]. Allergic reactions to foods, spices, and herbs may present with overlapping cutaneous and systemic manifestations, highlighting the importance of a detailed clinical history and careful identification of potential triggers [22]. Type 2 immune pathways are also implicated in allergic and fibroinflammatory disorders, including IgG4-related disease, illustrating the overlap between allergic inflammation and broader immune-mediated conditions [23]. Advanced disease may cause cytopenias, hepatosplenomegaly, lymphadenopathy, malabsorption, ascites, and progressive organ dysfunction [1,2,3,4,5,6,7,8].

The diagnosis of SM requires integration of clinical findings, serum tryptase concentrations, tissue histopathology, bone marrow morphology, immunophenotyping, and molecular testing [1,2,3,4,5,6,7,8]. Aberrant expression of CD25 and/or CD2 and detection of activating *KIT* mutations provide important diagnostic evidence. However, mast-cell burden, serum tryptase concentrations, mediator-related manifestations, and clinical aggressiveness do not always correlate [3,4,5,6,7,8].

Advanced SM includes aggressive systemic mastocytosis, systemic mastocytosis with an associated hematologic neoplasm (SM-AHN), and mast cell leukemia (MCL) [1,2,3,4,5,6,7,8]. MCL is a rare and highly aggressive form characterized by extensive involvement of the bone marrow and, in some cases, peripheral blood. Its diagnosis may be challenging because of morphological and immunophenotypic overlap with other myeloid malignancies, while treatment remains difficult despite advances in targeted therapy [24,25,26,27,28,29].

Real-world case series can provide valuable insights into the diagnostic challenges and biological diversity of SM, particularly when detailed morphological, immunophenotypic, and molecular findings are available [5,6,7,8,30,31,32,33,34,35,36].

Accordingly, this single-center study aimed to characterize the clinical, morphological, immunophenotypic, biochemical, and molecular heterogeneity of SM across a spectrum ranging from non-advanced disease to mast cell leukemia, including a case with a concomitant *FIP1L1::PDGFRA*-positive myeloid/lymphoid neoplasm with eosinophilia.

## 2. Materials and Methods

### 2.1. Study Design and Patient Selection

This retrospective, single-center observational study included six patients with systemic mastocytosis (SM) evaluated and managed at Sibiu County Emergency Clinical Hospital, Romania, through multidisciplinary collaboration among hematologists, allergists/immunologists, and dermatologists.

Patients were diagnosed and managed between 2018 and 2022. Follow-up extended from diagnosis to the most recent available assessment for surviving patients or to death for deceased patients. Of eight potentially eligible patients, six were included. One was excluded because the case had previously been published [37], and another was excluded because of insufficient clinical documentation following loss to follow-up.

Medical records were reviewed for demographic characteristics, clinical presentation, diagnostic history, organ involvement, laboratory findings, skin and bone marrow histopathology, immunophenotype, molecular abnormalities, treatment, and outcomes.

Given the small cohort and variable availability of historical investigations, analyses were performed descriptively at the individual-patient level. Missing data were recorded as unavailable and were not imputed.

### 2.2. Clinical and Laboratory Assessment

Clinical assessment included cutaneous, gastrointestinal, skeletal, hematologic, and constitutional manifestations, as well as hepatosplenomegaly, lymphadenopathy, and other evidence of organ involvement. Previous diagnoses and diagnostic delays were documented when available.

Laboratory investigations included complete blood counts, differential leukocyte counts, routine biochemical parameters, and serum total tryptase concentrations. Additional tests, including liver function parameters, vitamin B12, calcium, vitamin D, parathyroid hormone, ferritin, and coagulation studies, were reviewed when available.

Serum tryptase concentrations were interpreted using the laboratory reference threshold of 11 μg/L and assessed alongside clinical, histopathological, immunophenotypic, and molecular findings rather than as an isolated indicator of disease severity.

Imaging investigations included abdominal ultrasonography, computed tomography (CT), skeletal radiography, and dual-energy X-ray absorptiometry (DXA), according to clinical indications. Upper gastrointestinal endoscopy and mucosal biopsy were performed in selected patients.

### 2.3. Bone Marrow, Histopathological, and Immunophenotypic Assessment

Bone marrow evaluation included aspirate cytology, biopsy, histopathology, immunohistochemistry, and flow cytometry, according to the investigations available for each patient.

Morphological assessment focused on mast-cell infiltration, atypical spindle-shaped type I mast cells, immature or atypical type II forms, and the distribution and extent of marrow involvement. Histochemical staining included toluidine blue, Giemsa, and orthotoluidine, where available.

Immunophenotypic analysis assessed CD117, CD25, and CD2, with additional markers, including CD203c, CD34, CD123, HLA-DR, and CD30, evaluated when available. Flow cytometry was performed using a DxFLEX cytometer (Beckman Coulter) equipped with three lasers and supporting up to 13-color analysis.

Available cutaneous and gastrointestinal biopsy specimens were assessed using routine histopathology, special stains, and immunohistochemistry, as documented in the original diagnostic work-up.

### 2.4. Molecular Analysis

Molecular testing was performed according to the diagnostic investigations available for each patient. Where detailed laboratory reports were accessible, *KIT* D816V was assessed using targeted allele-specific polymerase chain reaction (AS-PCR) rather than comprehensive *KIT* sequencing.

In Patient 2, testing was performed on bone marrow and peripheral blood, with an analytical sensitivity of approximately 1–3%. In Patients 3–5, peripheral blood testing used AS-PCR with a reported detection limit of approximately 1%. Detailed methodological information was unavailable for Patients 1 and 6.

In Patient 5, the *FIP1L1::PDGFRA* fusion transcript was investigated in peripheral blood using reverse transcription followed by nested polymerase chain reaction (PCR).

Quantitative *KIT* D816V variant allele frequency (VAF) was not systematically available. Under the 2022 World Health Organization (WHO) classification, a *KIT* D816V VAF ≥ 10% in peripheral blood or bone marrow constitutes a B-finding indicating increased disease burden. The absence of quantitative VAF measurements therefore limited retrospective assessment according to contemporary criteria.

Patients were originally diagnosed using the WHO criteria applicable during the study period. Diagnosis required either the major criterion and at least one minor criterion or at least three minor criteria. The major criterion consisted of multifocal dense infiltrates of ≥15 mast cells in bone marrow or another extracutaneous organ. Minor criteria included (i) more than 25% atypical or spindle-shaped mast cells; (ii) an activating *KIT* mutation at codon 816; (iii) aberrant CD25 and/or CD2 expression; and (iv) persistent serum total tryptase > 20 ng/mL, except in patients with an associated myeloid neoplasm.

Disease subclassification relied on B- and C-findings according to the applicable WHO framework. Prognostic assessment primarily considered disease subtype, organ damage, and available clinical and laboratory parameters.

Next-generation sequencing (NGS) was not routinely performed because it was not reimbursed by the national health insurance system during the study period and represented a substantial financial burden. Although reimbursement has subsequently become available for patients with leukemias and myeloproliferative neoplasms, both patients with advanced hematologic disease had died before additional molecular profiling could be undertaken.

### 2.5. Treatment and Outcome Assessment

Treatment information was retrospectively extracted from medical records and included symptomatic anti-mediator therapy, hypomethylating agents and imatinib in Patient 6 with mast cell leukemia, and midostaurin and other targeted treatments in Patient 5 with *KIT* D816V-positive SM and a concomitant *FIP1L1::PDGFRA*-positive myeloid/lymphoid neoplasm with eosinophilia.

Supportive care and other disease-directed interventions were also recorded. Because treatment strategies differed according to disease subtype, age, comorbidities, and treatment availability, therapeutic exposure was assessed descriptively rather than comparatively.

Documented outcomes included symptom control, disease stability or progression, treatment-related complications, hospitalizations, and mortality. Formal survival analyses were not performed because follow-up duration varied across patients.

### 2.6. Statistical Analysis

Given the small sample size and clinical heterogeneity, statistical analysis was descriptive. Continuous variables were summarized using medians and ranges, while categorical variables were presented as absolute numbers and proportions, where appropriate.

No inferential statistical tests, regression analyses, or correlation assessments were performed. The analysis focused on individual clinical characteristics, organ involvement, laboratory and molecular findings, treatment, and outcomes.

### 2.7. Ethical Considerations

All patient data were anonymized to protect confidentiality. Details regarding ethical approval and informed consent are provided in the corresponding statements at the end of the manuscript.

Written informed consent for the use of clinical and pathological data for research purposes was obtained from all patients or their legal guardians before inclusion. All data were fully anonymized to protect patient confidentiality.

## 3. Results

### 3.1. Cohort Characteristics

The study included six patients with systemic mastocytosis (SM), four males and two females, aged 16–86 years (median age, 45.5 years). Their demographic, clinical, and diagnostic characteristics are summarized in Table 1.

Patients 1–4 had originally been classified as having smoldering systemic mastocytosis (SSM). However, because the retrospective records did not consistently document at least two B-findings, these patients were conservatively classified as having non-advanced SM according to contemporary criteria.

The International Prognostic Scoring System for Mastocytosis (IPSM) was retrospectively applied to the four patients with non-advanced SM. All were younger than 60 years. Patients 1 and 4 had alkaline phosphatase (ALP) concentrations ≥ 100 U/L and were classified as intermediate-1 risk, whereas Patients 2 and 3 were classified as low risk.

For the two patients with advanced hematologic disease, prognostic assessment was performed cautiously because of their distinct molecular and clinical characteristics. Patient 5 had a *FIP1L1::PDGFRA*-positive myeloid/lymphoid neoplasm with eosinophilia and prominent systemic mast-cell involvement. Although several IPSM risk factors were present, the applicability of the advanced SM prognostic model to this molecularly defined neoplasm requires caution. Therefore, no definitive IPSM risk category was assigned.

Patient 6, diagnosed with mast cell leukemia, had an IPSM score of 3, corresponding to the AdvSM-3 risk category. Nevertheless, the rapidly progressive clinical course highlighted the limitations of prognostic assessment based solely on conventional clinical and laboratory parameters.

The Mutation-Adjusted Risk Score (MARS) could not be calculated because comprehensive next-generation sequencing data were unavailable.

Bone marrow involvement was documented in all patients, whereas gastrointestinal, hepatosplenic, skeletal, and hematologic manifestations varied considerably. Patient 5 had a *FIP1L1::PDGFRA*-positive myeloid/lymphoid neoplasm with eosinophilia and prominent systemic mast-cell involvement. Patient 6 presented with MCL and a rapidly progressive hematologic course.

Baseline serum tryptase concentrations ranged from 15.8 to 348 μg/L. The highest concentration occurred in Patient 5, whereas Patient 6 had a comparatively modest elevation of 28.5 μg/L despite extensive marrow and peripheral blood involvement.

Overall, the cohort illustrates the broad clinicopathological spectrum of SM, from predominantly cutaneous, non-advanced disease to advanced phenotypes associated with multiorgan dysfunction and aggressive hematologic involvement.

### 3.2. Diagnostic Pathways and Initial Diagnostic Challenges

Diagnostic pathways differed considerably across the cohort, often involving multiple specialties before systemic mastocytosis (SM) was recognized. In several patients, cutaneous manifestations preceded diagnosis by years and were initially attributed to more common dermatological conditions.

**Figure 1:**
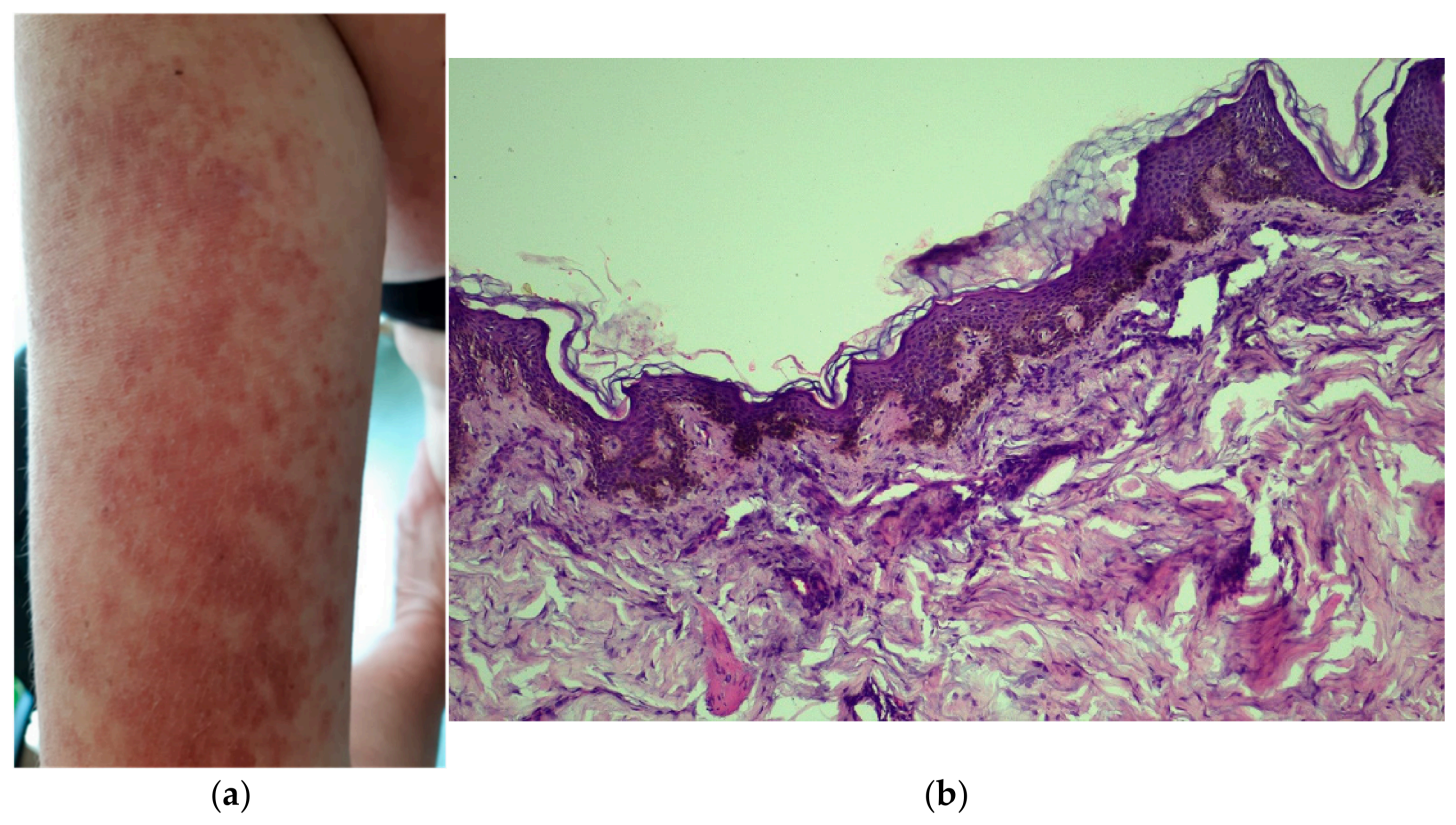
(**a**) Extensive telangiectatic macules (trunk and extensor surface of the limbs). (**b**) Characteristic perivascular and diffuse interstitial mast cell infiltrates (toluidine blue and Giemsa stains).

Patient 1, a 54-year-old female diagnosed in 2018, experienced a diagnostic delay of approximately 3–4 years. She presented with intermittently pruritic telangiectatic macules involving the trunk and extensor surfaces of the limbs (Figure 1a). Initial diagnoses included acute urticaria, vascular purpura, and telangiectasia macularis eruptiva perstans (TMEP). However, the initial skin biopsy, performed in May 2018, was inconclusive on routine hematoxylin and eosin staining. Recurrent gastrointestinal symptoms, including nausea and epigastric discomfort, were associated with endoscopically documented erosive gastroduodenitis. The prolonged diagnostic process was accompanied by a documented secondary depressive episode.

Following multidisciplinary evaluation, re-examination of the skin biopsy using toluidine blue and Giemsa stains revealed characteristic perivascular and interstitial mast-cell infiltrates (Figure 1b). Bone marrow examination identified 5–6% atypical mast cells overall, reaching 42% in marrow particle aggregates. Additional findings included aberrant CD117+/CD25+ expression, elevated serum tryptase (105 ng/mL), and the *KIT* D816V mutation in peripheral blood, confirming SM. This case illustrates the diagnostic limitations of routine histopathology without mast-cell-specific staining.

Patient 2, a 27-year-old male diagnosed in 2020, presented with thoracoabdominal telangiectatic macules and a pigmented maculopapular eruption. Skin histopathology revealed basal epidermal hyperpigmentation and perivascular and interstitial infiltrates containing oval-to-elongated cells with eosinophilic cytoplasmic granules and occasional eosinophils. Immunohistochemistry demonstrated strong, diffuse CD117 positivity. These findings, together with serum tryptase evaluation, supported cutaneous mastocytosis (urticaria pigmentosa), prompting further systemic investigation.

**Figure 2:**
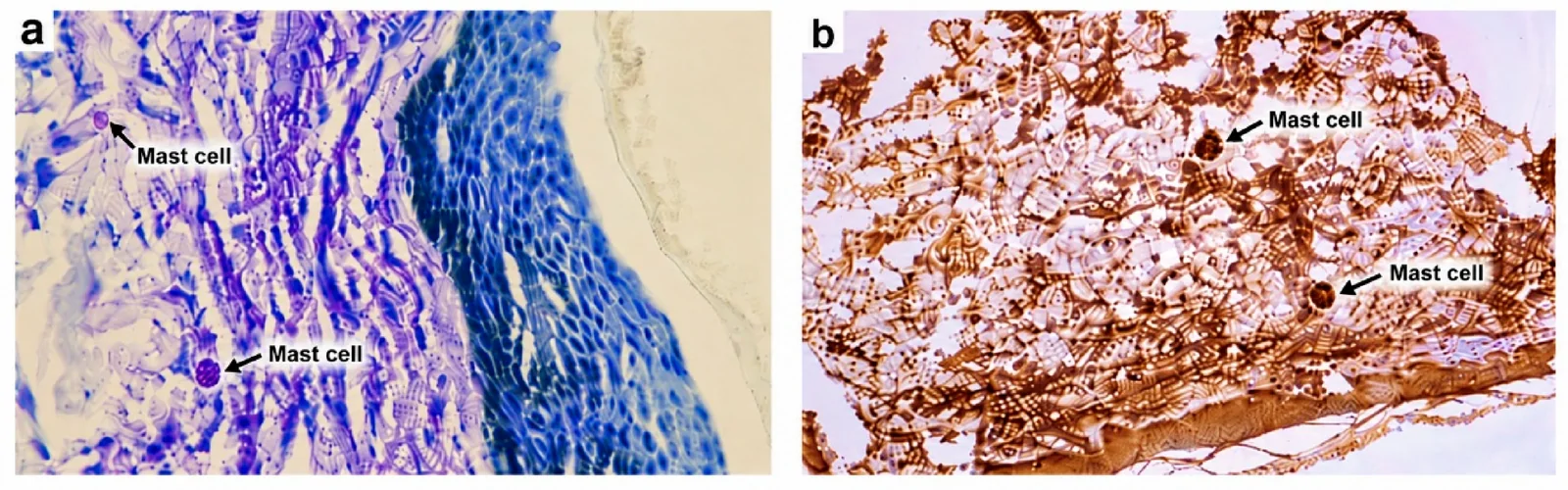
(**a**) Moderate inflammatory infiltrate arranged both perivascular and “in-band”, composed of mast cells, some degranulated and lymphocytes (Giemsa and toluidine blue stains). (**b**) Basophilic granules and immunohistochemical staining showing CD117-positive mast cells.

Patient 3, a 37-year-old male diagnosed in 2022, presented with a pink–violaceous maculopapular eruption suggestive of mastocytosis. Skin biopsy demonstrated predominantly perivascular and band-like dermal mast-cell infiltrates, including degranulated cells, accompanied by lymphocytes and interstitial edema. Giemsa and toluidine blue staining highlighted cytoplasmic granules, while immunohistochemistry confirmed CD117-positive mast cells (Figure 2a,b). Subsequent bone marrow examination and immunophenotyping demonstrated abnormal mast-cell infiltration, and molecular testing identified *KIT* D816V.

**Figure 3:**
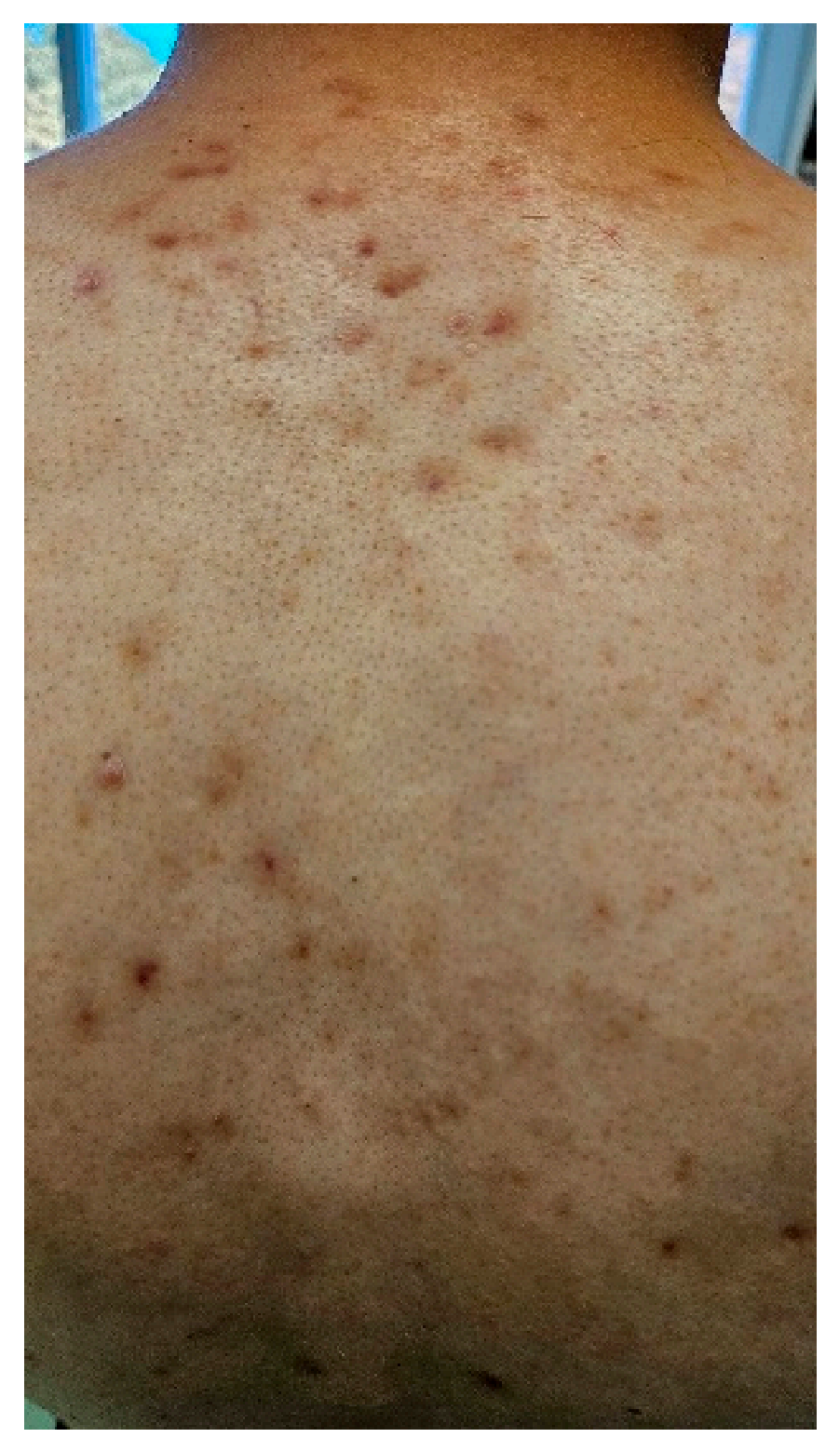
Pink–violaceous maculopapular rash with cicatricial aspect on dorsal trunk.

Patient 4, a 16-year-old male evaluated in 2021, developed disseminated, non-pruritic, acne-like cutaneous lesions in December 2020 that were unresponsive to topical treatment (Figure 3). Their appearance caused considerable psychosocial distress and progressive social withdrawal. His family history included maternal colorectal cancer with hepatic metastases and acute myeloid leukemia in a maternal aunt, who subsequently died from transplantation-related complications. Laboratory assessment revealed mildly elevated serum tryptase (23.1 μg/L), and molecular testing identified *KIT* D816V. These findings prompted further evaluation to distinguish cutaneous mastocytosis from systemic disease with skin involvement.

**Figure 4:**
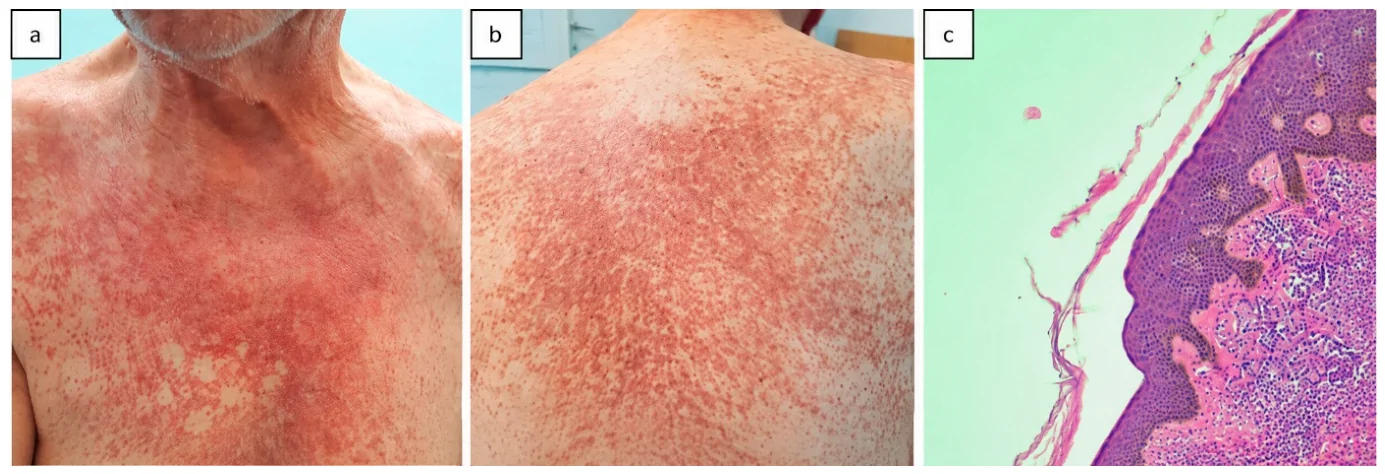
Patient 5. (**a**,**b**) Disseminated maculopapular rash with purpuric appearance. (**c**) Mast-cell infiltrate consisting of round-to-oval cells with slightly basophilic cytoplasm and hyperchromatic nuclei, without mitoses.

Patient 5, a 79-year-old male diagnosed in 2021, had a 2-year history of a pruritic eruption initially considered vascular or senile purpura. Persistent symptoms prompted serum tryptase testing, which revealed concentrations exceeding 300 μg/L. Examination showed confluent maculopapular lesions with a purpuric appearance (Figure 4a,b), hepatomegaly (16 cm), and splenomegaly (17 cm). Gastrointestinal endoscopy identified a gastric ulcer, a large sliding hiatal hernia, and erosive gastritis. Constitutional symptoms included marked fatigue, weakness, anorexia, and unintentional weight loss. Skin histopathology, supported by toluidine blue staining, confirmed cutaneous mastocytosis, while the systemic findings prompted comprehensive hematologic assessment.

Patient 6, an 86-year-old female diagnosed in 2018, presented with fever, chills, wheezing, diaphoresis, and dyspnea. Her medical history included type 2 diabetes mellitus and arterial hypertension. The clinical course rapidly progressed to generalized edema, pleural and pericardial effusions, ascites, and cardiorespiratory failure. Detailed biochemical and cytological characterization of the effusions was unavailable. Hematologic evaluation revealed leukocytosis, anemia, numerous circulating blasts, and extensive bone marrow infiltration. The initial differential diagnosis included acute mast-cell and basophilic leukemia. Subsequent immunophenotypic analysis supported mast cell leukemia (MCL).

### 3.3. Clinical Manifestations and Organ Involvement

Clinical manifestations varied considerably across the cohort. Cutaneous involvement was documented in five of the six patients and included telangiectatic macules and disseminated maculopapular eruptions, occasionally associated with pruritus or a purpuric appearance.

Gastrointestinal manifestations were documented in several patients. Patient 1 reported nausea and abdominal discomfort, with endoscopy revealing erosive gastroduodenitis; gastric mast-cell infiltration was not documented. In Patient 3, gastric biopsies showed chronic gastritis with moderate atrophy and mild inflammatory activity, without *Helicobacter pylori*. Patient 5 had histologically confirmed gastric mucosal mast-cell infiltration in the context of advanced multiorgan disease.

**Figure 5:**
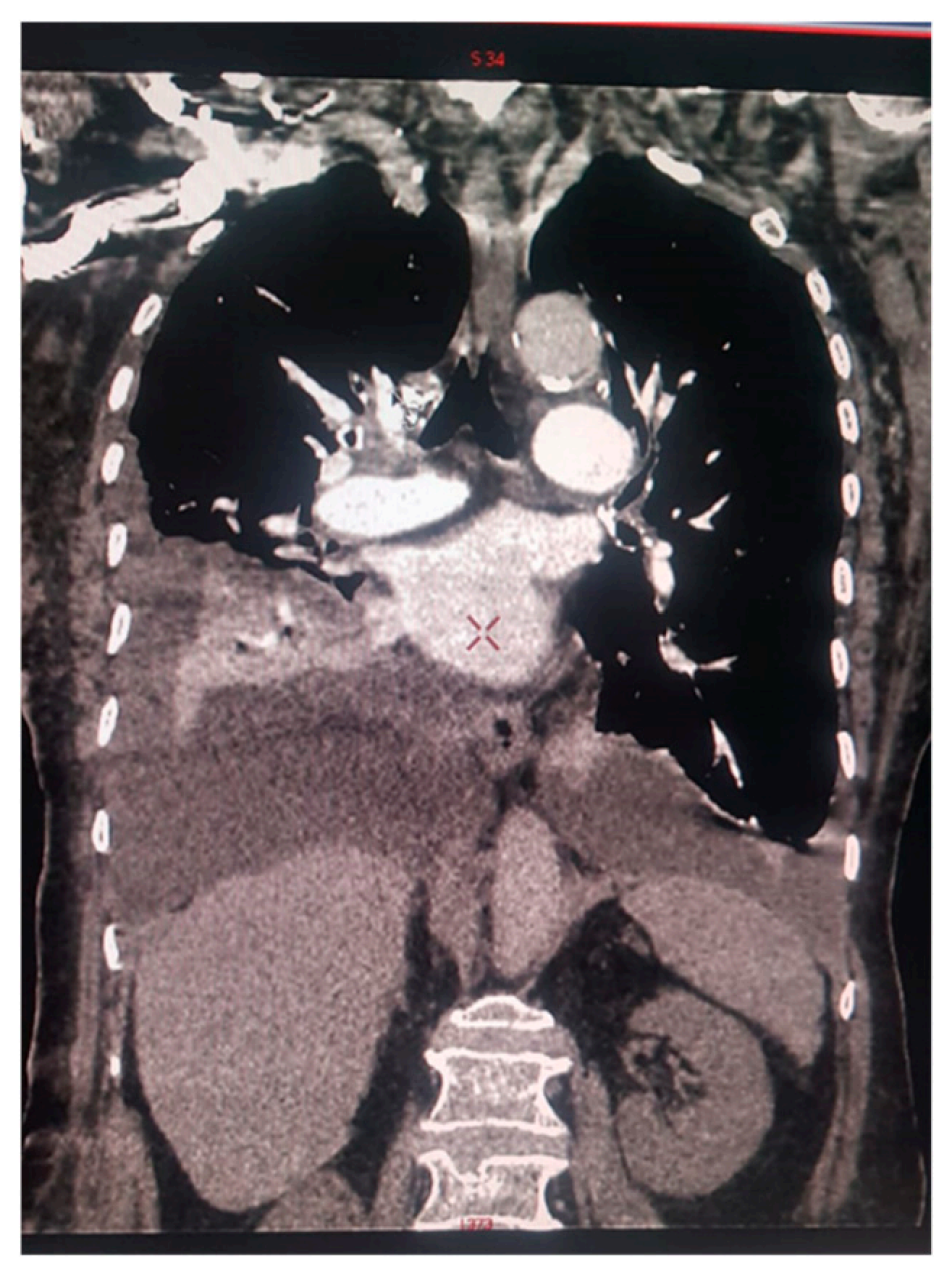
Patient 6. Computed tomography (CT) image at the time of diagnosis. The image is provided for illustrative purposes; the relevant radiological findings are described in the main text.

Hepatosplenic involvement was more prominent in patients with extensive systemic disease. Patient 4 had moderate hepatosplenomegaly on computed tomography (CT). Patient 5 presented with hepatosplenomegaly, lymphadenopathy, constitutional symptoms, and weight loss. Thoracoabdominopelvic CT demonstrated hepatomegaly (16 cm), splenomegaly (17 cm) with a 3 cm focal nodule, celiacomesenteric and retrocrural lymphadenopathy (up to 2 cm), and diffuse mixed osteolytic and osteosclerotic vertebral lesions. Patient 6 developed pleural and pericardial effusions, ascites, and hepatomegaly of potentially multifactorial origin, including suspected mast-cell infiltration and passive hepatic congestion (Figure 5).

**Figure 6:**
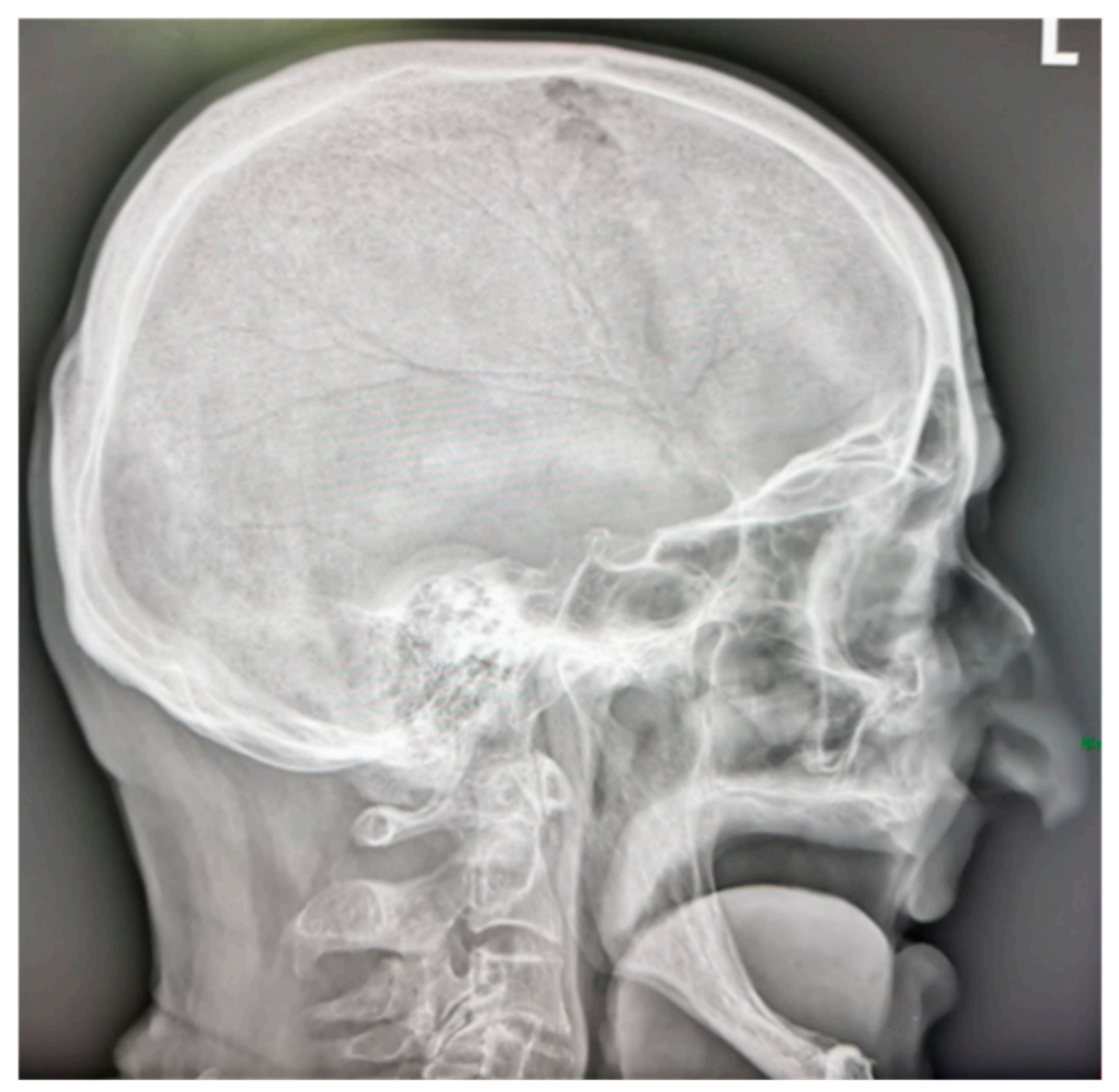
Patient 5. Skeletal radiography of the skull revealed a 1.7 cm radiolucent area in the frontoparietal region, consistent with an osteolytic lesion, alongside adjacent structural alterations.

Skeletal involvement was heterogeneous. Patients 1–4 underwent bone mineral density assessment by dual-energy X-ray absorptiometry (DXA). Clinically significant osteoporosis requiring antiresorptive treatment was documented only in Patient 1. Patient 2 sustained a traumatic knee ligament rupture, while Patient 4 had vertebral abnormalities involving T10–T11. Patient 5 exhibited diffuse thoracolumbar structural changes, including areas of bone rarefaction and sclerosis, together with a 1.7 cm osteolytic skull lesion (Figure 6).

Overall, organ involvement ranged from predominantly cutaneous manifestations in non-advanced SM to extensive gastrointestinal, hepatosplenic, skeletal, and hematologic abnormalities in patients with advanced disease.

### 3.4. Laboratory Profiles, Biomarker Evaluation, Bone Marrow Morphology, and Immunophenotypic Findings

Routine hematologic parameters were largely unremarkable in Patients 1–4, whereas Patients 5 and 6 showed substantial abnormalities.

Patient 5 had marked leukocytosis (27.51 × 10^9^/L), mild normocytic anemia (hemoglobin 11.3 g/dL), and a preserved platelet count (229 × 10^9^/L). The peripheral blood differential demonstrated neutrophilia, monocytosis, and eosinophilia, together with circulating immature granulocytes. Lactate dehydrogenase was within normal limits, and moderate inflammatory abnormalities were documented. Sputum culture was positive for *Klebsiella oxytoca*.

Patient 6 presented with leukocytosis (19.10 × 10^9^/L), normocytic anemia (hemoglobin 9.5 g/dL), and a preserved platelet count (206 × 10^9^/L). Peripheral blood examination revealed 51% circulating blasts and 2% erythroblasts, together with immature granulocytic forms, consistent with a leukemic presentation.

Bone marrow findings ranged from focal atypical mast-cell infiltrates in non-leukemic cases to extensive leukemic involvement. Patients 1–4 predominantly exhibited spindle-shaped type I mast cells, with variable proportions of atypical type II forms. The main morphological, immunophenotypic, and molecular findings are summarized in Table 2.

**Figure 7:**
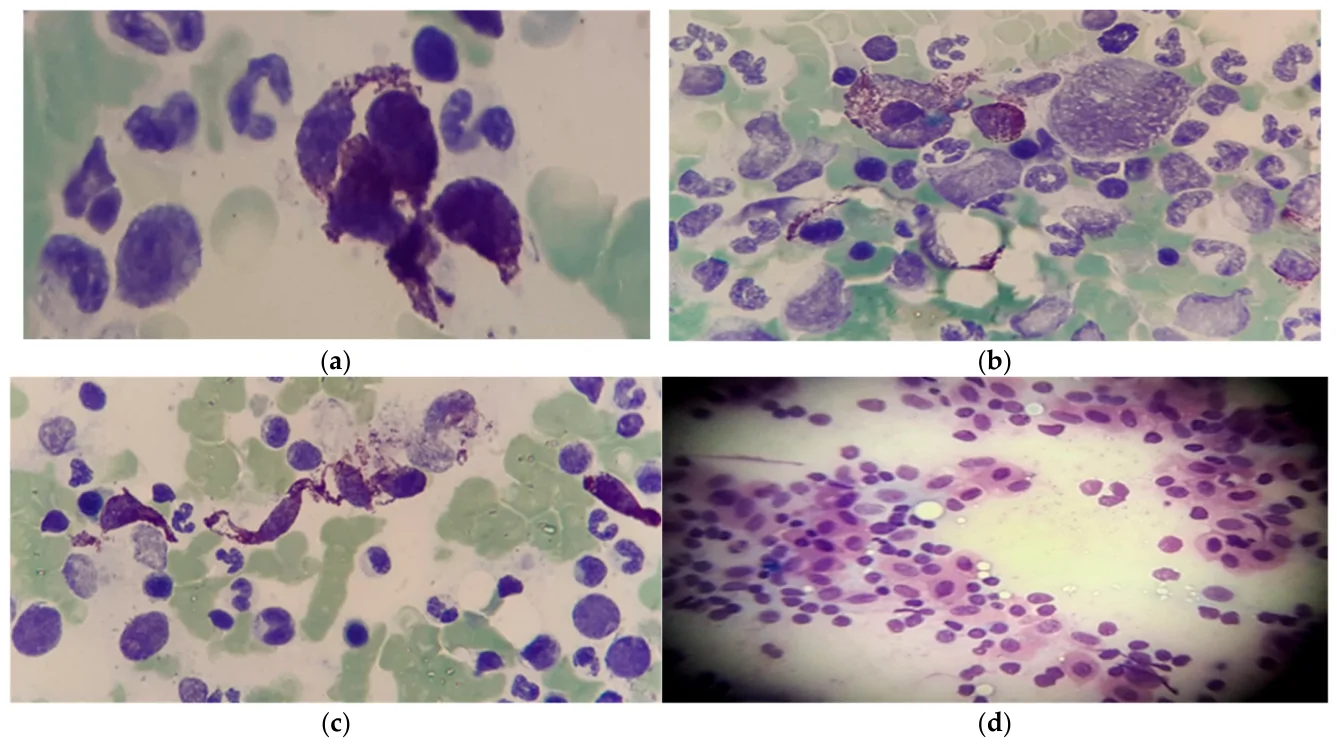
(**a**–**d**) Bone marrow findings in Patient 1. Bone marrow aspirate and histopathological examination showing atypical mast-cell infiltration, including nodular aggregates. Approximately 5–6% atypical mast cells were identified overall, with higher proportions in marrow particles, reaching up to 42%. Mast cells were highlighted by orthotoluidine, Giemsa, and toluidine blue staining, and immunophenotyping demonstrated an aberrant CD117+/CD25+ mast-cell population.

In Patient 1, bone marrow examination revealed approximately 5–6% atypical mast cells overall, reaching 42% in marrow particle aggregates. Nodular infiltrates and aberrant CD117+/CD25+ expression supported clonal mast-cell disease (Figure 7a–d).

Patient 2 had approximately 3% atypical spindle-shaped mast cells in myeloid tissue and 8–15% in marrow particles, with focal aggregates reaching 50%. Flow cytometry identified an aberrant CD117++/CD25+/CD2+ mast-cell population.

Patient 3 showed predominantly type I mast cells, accounting for approximately 2–5% of myeloid tissue and up to 15% of marrow particles. The abnormal population expressed CD117, CD203c, CD25, and CD2, with variable or weak HLA-DR, CD34, and CD123 expression.

Patient 4 exhibited type I and type II mast cells, representing approximately 1–3% of myeloid tissue and 13% of marrow particles. Focal mast-cell infiltration and suggestive flow-cytometric findings prompted a second-opinion review and repeat bone marrow biopsy, which confirmed SM.

**Figure 8:**
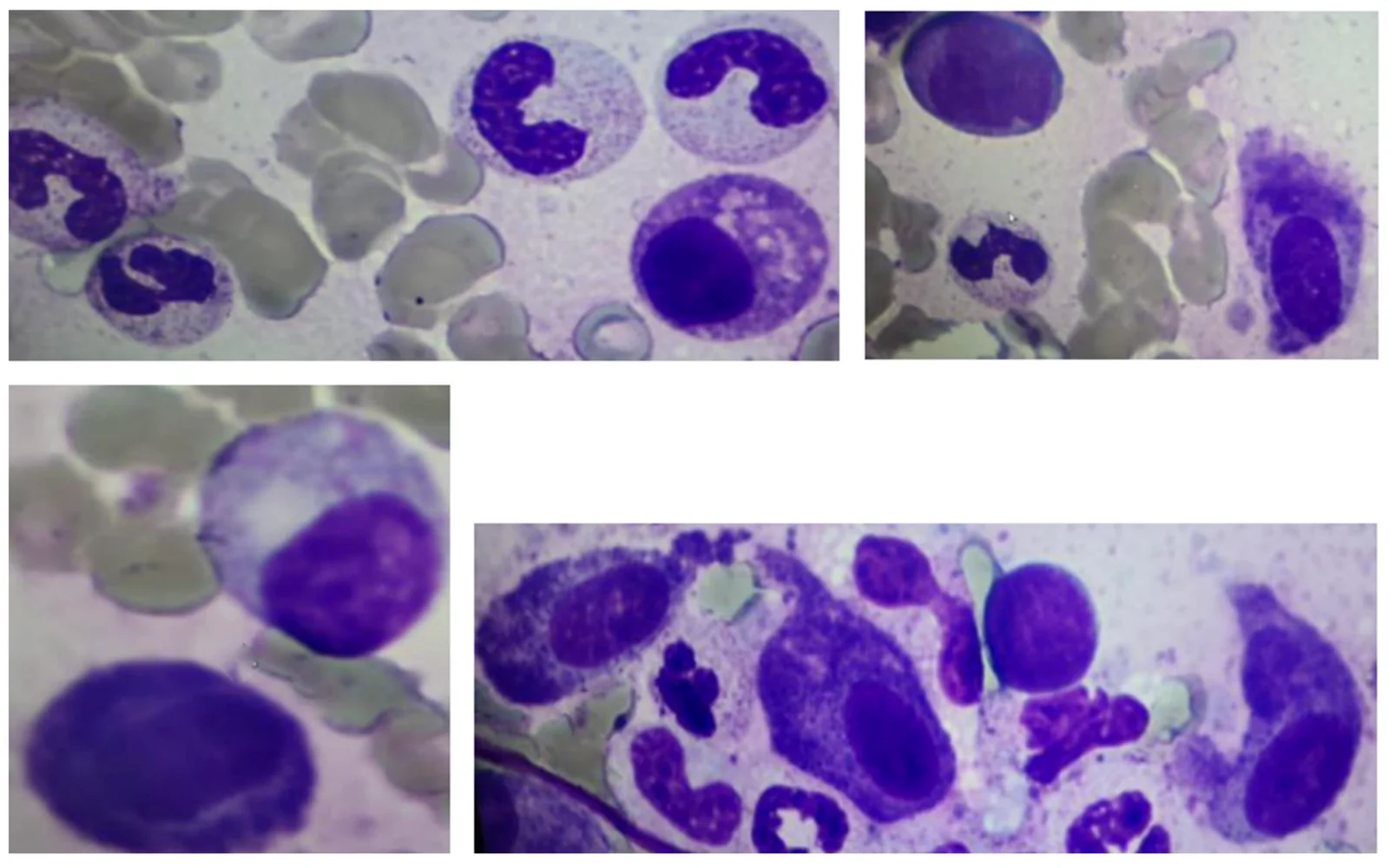
Representative bone marrow aspirate findings in Patient 5. The marrow showed mast-cell and granulocytic hyperplasia, with atypical mast cells unevenly distributed and exceeding 25% in some marrow particles. Associated eosinophilic hyperplasia was also present, with eosinophils accounting for approximately 10% of marrow cells. Immunophenotyping identified a clonal mast-cell population expressing CD117, CD45, CD203c, CD25, and CD2.

Patient 5 had mast-cell and granulocytic hyperplasia, with mast-cell proportions exceeding 25% in some marrow particles and eosinophils accounting for approximately 10% of marrow cells. Immunophenotyping demonstrated CD117, CD45, CD203c, CD25, and CD2 expression (Figure 8).

**Figure 9:**
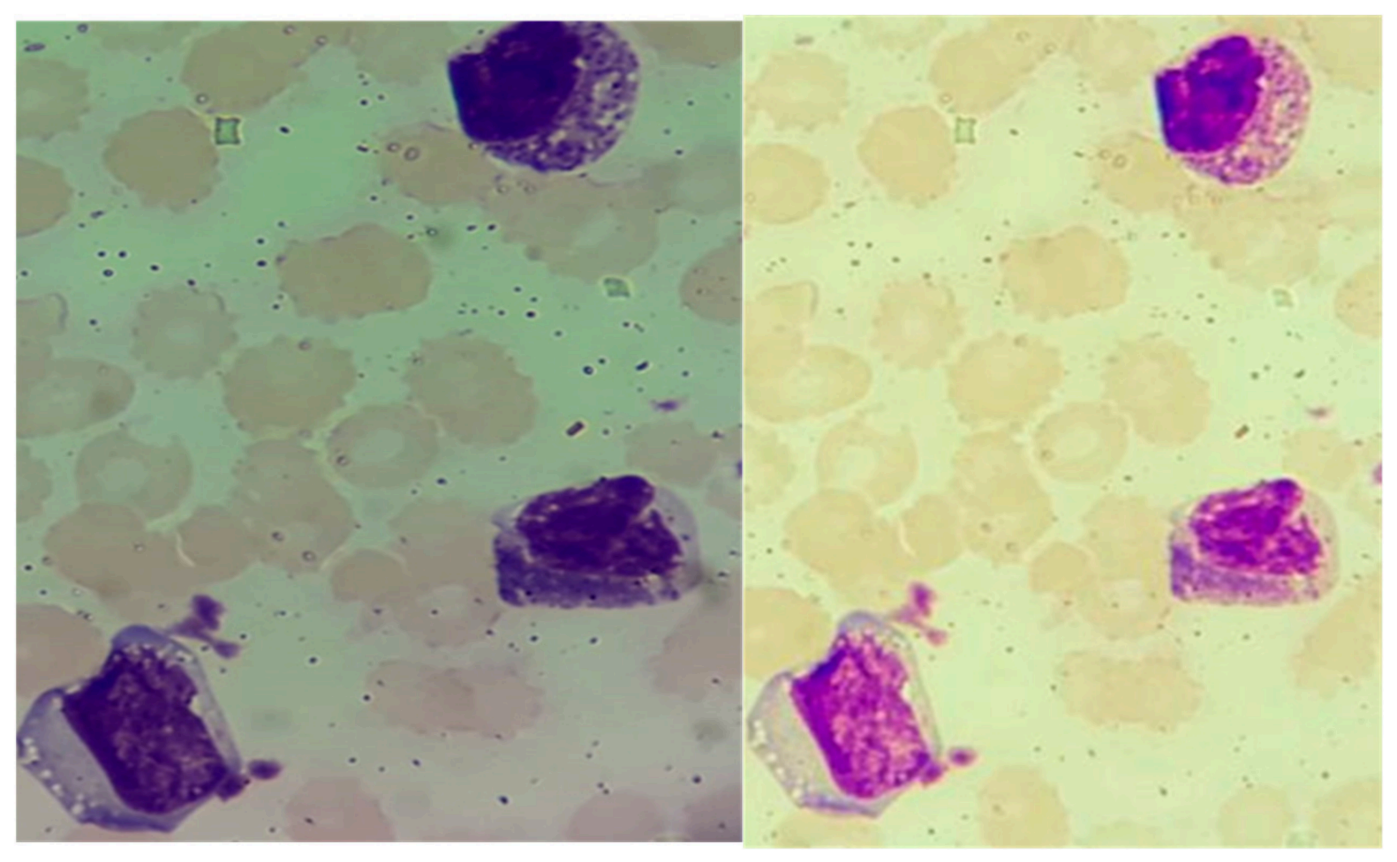
Bone marrow findings in Patient 6 with mast cell leukemia. The marrow was markedly hypercellular, with approximately 65% blast-like cells on aspirate and extensive leukemic infiltration. The abnormal cells showed immature morphology, with medium-sized cells, round-to-oval nuclei, visible nucleoli, and basophilic cytoplasm; toluidine blue staining highlighted metachromatic granules in a substantial proportion of cells. These findings, together with the immunophenotypic profile, supported mast-cell-lineage differentiation.

Patient 6 displayed markedly hypercellular bone marrow with approximately 65% blast-like cells. These cells had round-to-oval nuclei, visible nucleoli, basophilic cytoplasm, and occasional cytoplasmic inclusions. Increased basophilic precursors and mature basophils were also observed. Toluidine blue staining was positive in approximately 30% of marrow cells, raising the differential diagnosis of acute basophilic leukemia and mast cell leukemia (Figure 9).

**Figure 10:**
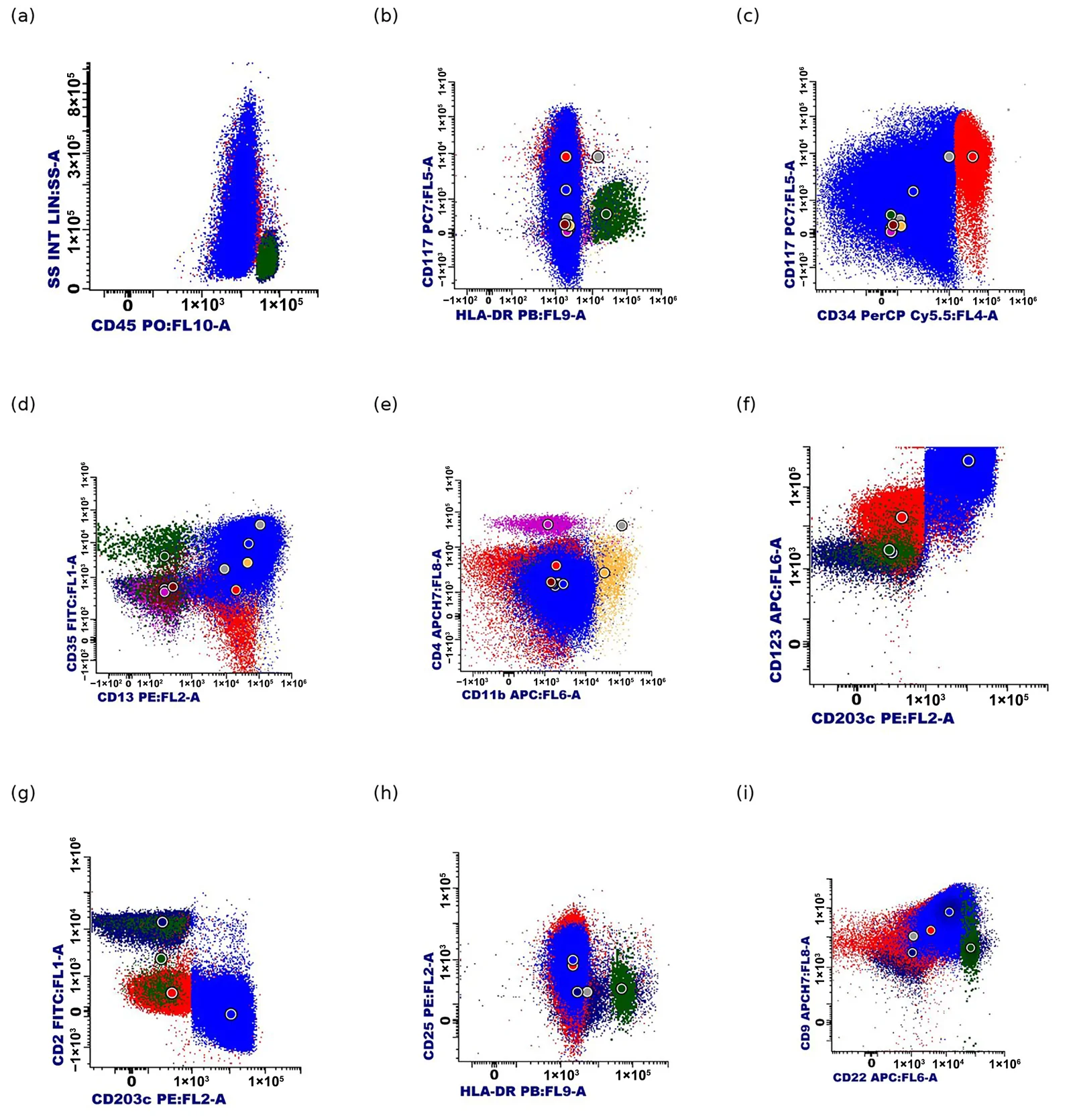
Flow-cytometric immunophenotype of bone marrow cells in Patient 6 with mast cell leukemia. Nine representative flow-cytometric plots (**a**–**i**) illustrate two abnormal immature cell populations with immunophenotypic features suggestive of mast-cell-lineage differentiation. Subpopulation 1 (30%) exhibited strong CD117 expression, with absent HLA-DR and CD123 expression. Subpopulation 2 (50%) showed CD117, HLA-DR, CD123, and CD203c expression. Both populations expressed CD34 and lacked CD2, with variable CD25 expression. The different colors of the dots reflect the original flow-cytometry acquisition and display settings and should not be interpreted as consistent identifiers of the same cell populations across all panels.

Flow cytometry identified two abnormal immature cell populations with immunophenotypic features suggestive of mast-cell-lineage differentiation. Subpopulation 1 (30%) showed CD45dim, CD34+, CD117++, CD13dim, HLA-DR−, CD38−, CD123−, CD2−, and variable CD25, CD56, CD9, and CD15 expression. Subpopulation 2 (50%) expressed CD34, CD117, CD13, HLA-DR, CD123, CD203c, CD35, CD22dim, and CD9, with absent CD38, CD56, and CD2 and variable CD25 expression. These findings were interpreted as two distinct stages of mast-cell-lineage maturation and supported the diagnosis of mast cell leukemia (Figure 10).

These findings were interpreted in conjunction with bone marrow morphology and the overall clinicopathological presentation. The plots represent the original flow-cytometric analysis. Cell colors reflect the display settings used during data acquisition and should not be interpreted as uniform identifiers of the two abnormal subpopulations across all panels.

Overall, bone marrow findings demonstrated substantial morphological and immunophenotypic heterogeneity, ranging from focal clonal mast-cell infiltration to extensive immature leukemic involvement.

### 3.5. Serum Tryptase, KIT Status, and Molecular Findings

**Figure 11:**
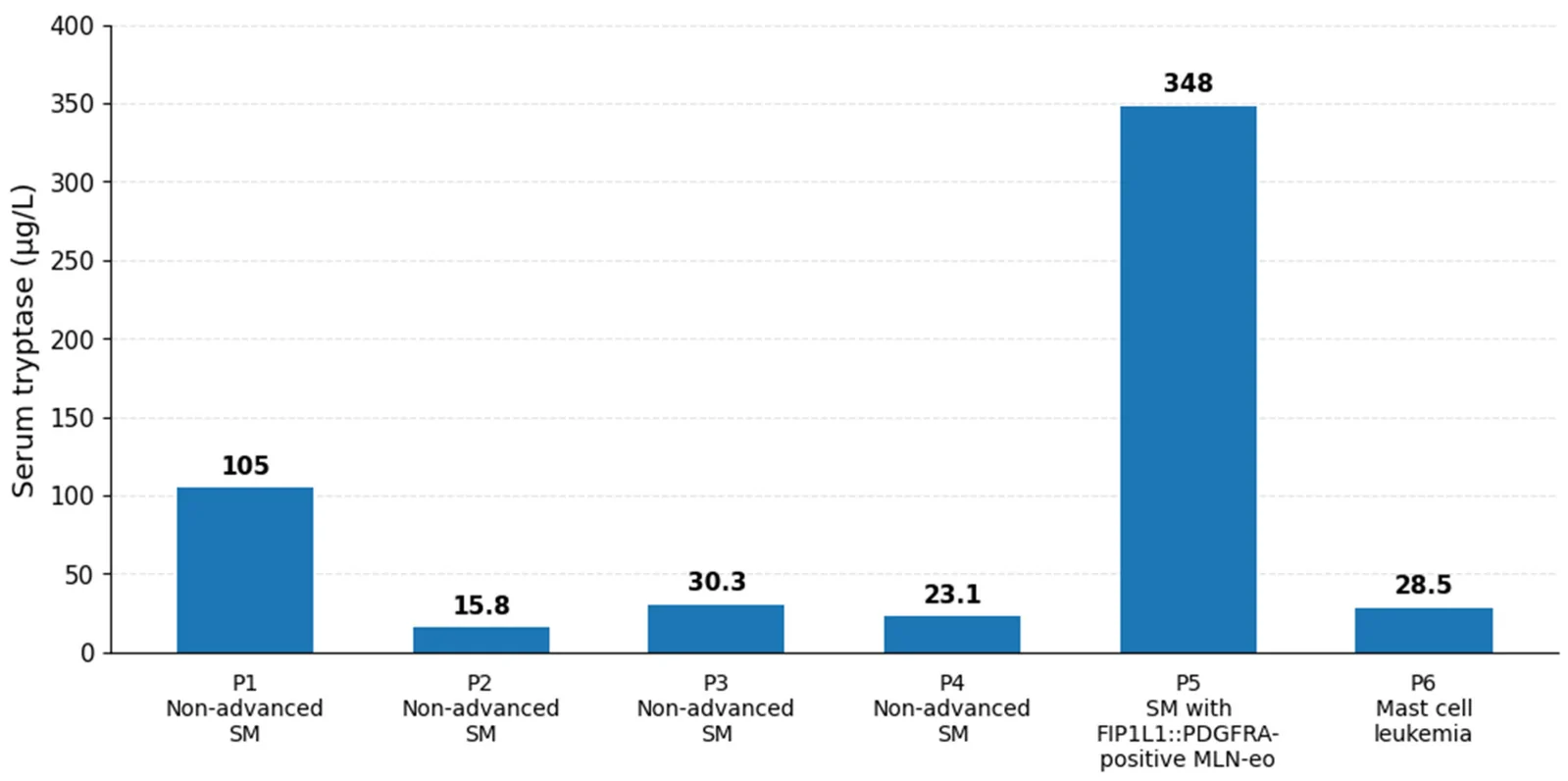
Serum tryptase concentrations across the six patients with systemic mastocytosis.

Serum tryptase concentrations varied considerably across the cohort, ranging from 15.8 to 348 μg/L. The highest value was recorded in Patient 5 (348 μg/L), who had a *FIP1L1::PDGFRA*-positive myeloid/lymphoid neoplasm with eosinophilia, prominent systemic mast-cell involvement, and extensive multiorgan disease. In contrast, Patient 6, diagnosed with mast cell leukemia (MCL), had a substantially lower concentration (28.5 μg/L) despite extensive bone marrow and peripheral blood involvement. These findings illustrate the discordance between serum tryptase concentrations and clinical aggressiveness in this cohort (Figure 11).

Activating *KIT* mutations were detected in Patients 1–5, whereas Patient 6 tested negative for *KIT* D816V.

Additional molecular testing in Patient 5 identified the *FIP1L1::PDGFRA* fusion, consistent with the associated eosinophilic and myeloid phenotype. According to the 2022 World Health Organization (WHO) and International Consensus Classification (ICC) frameworks, this molecular alteration establishes the primary diagnosis of a myeloid/lymphoid neoplasm with eosinophilia and a defining *PDGFRA* rearrangement, accompanied in this case by prominent systemic mast-cell involvement.

In Patient 6, molecular testing for *FLT3*, *BCR::ABL1*, *NPM1*, *RUNX1::RUNX1T1* (AML1-ETO), and *CBFB* rearrangements was negative. The diagnosis of MCL was therefore supported primarily by the hematologic presentation, bone marrow morphology, and immunophenotypic findings rather than by an identified disease-defining molecular alteration.

### 3.6. Treatment and Clinical Outcomes

Treatment strategies varied according to disease phenotype, patient age, comorbidities, and therapeutic availability. Symptom-control measures, disease-modifying treatments, and clinical outcomes are summarized in Table 3.

Formal International Working Group–Myeloproliferative Neoplasms Research and Treatment (IWG-MRT) response categories could not be consistently assigned because the required parameters were incompletely documented. Therefore, treatment responses were described using the available clinical and hematologic findings.

Patients 1–4 received predominantly symptom-directed treatment and remained under clinical surveillance. Patient 1 was treated with sodium cromoglycate and famotidine. Because of persistent symptoms, imatinib (100 mg/day) was administered empirically for approximately three months but discontinued because of insufficient clinical benefit.

During the available follow-up, no progression to advanced systemic mastocytosis or development of new C-findings was documented in Patients 1–4. Patient 2 underwent orthopedic surgery without mast-cell-related complications, while Patients 3 and 4 remained clinically stable. Serial tryptase measurements and organ assessments were not consistently available, limiting evaluation of longitudinal disease changes.

The exact duration of follow-up and the dates of the last available clinical assessments could not be reliably established for Patients 1–4 from the retrospective medical records. Although the available documentation indicated clinical stability without recorded progression to advanced systemic mastocytosis, the absence of precise follow-up intervals limits the interpretation of their long-term outcomes.

Patient 5, who had a *FIP1L1::PDGFRA*-positive myeloid/lymphoid neoplasm with eosinophilia and prominent systemic mast-cell involvement, received midostaurin, sodium cromoglycate, famotidine, sucralfate, antibiotics, anticoagulation, and supportive care. Initial clinical improvement was observed, and additional imatinib therapy was considered for the *FIP1L1::PDGFRA*-positive component. However, the patient sustained a pertrochanteric fracture requiring surgery, which was completed without mast-cell-related perioperative complications. Subsequently, bronchopneumonia and cardiorespiratory deterioration necessitated intensive care, ultimately resulting in death.

Patient 6 received decitabine followed by decitabine combined with imatinib for four cycles. Treatment produced a transient reduction in leukocyte and circulating blast counts, accompanied by temporary improvement in ascites and cardiorespiratory symptoms. However, the response was not sustained, and the disease progressed with persistent circulating blasts and recurrent cardiopulmonary complications. The patient died in June 2018, approximately five months after diagnosis.

Overall, patients with non-advanced systemic mastocytosis remained clinically stable during documented follow-up, whereas both patients with advanced hematologic disease experienced severe complications and fatal outcomes. These cases also emphasize the importance of multidisciplinary management, particularly during surgical procedures, when individualized perioperative assessment and preventive strategies are essential to reduce the risk of mast-cell activation and associated complications [38,39].

### 3.7. Integrated Clinical Spectrum

**Figure 12:**
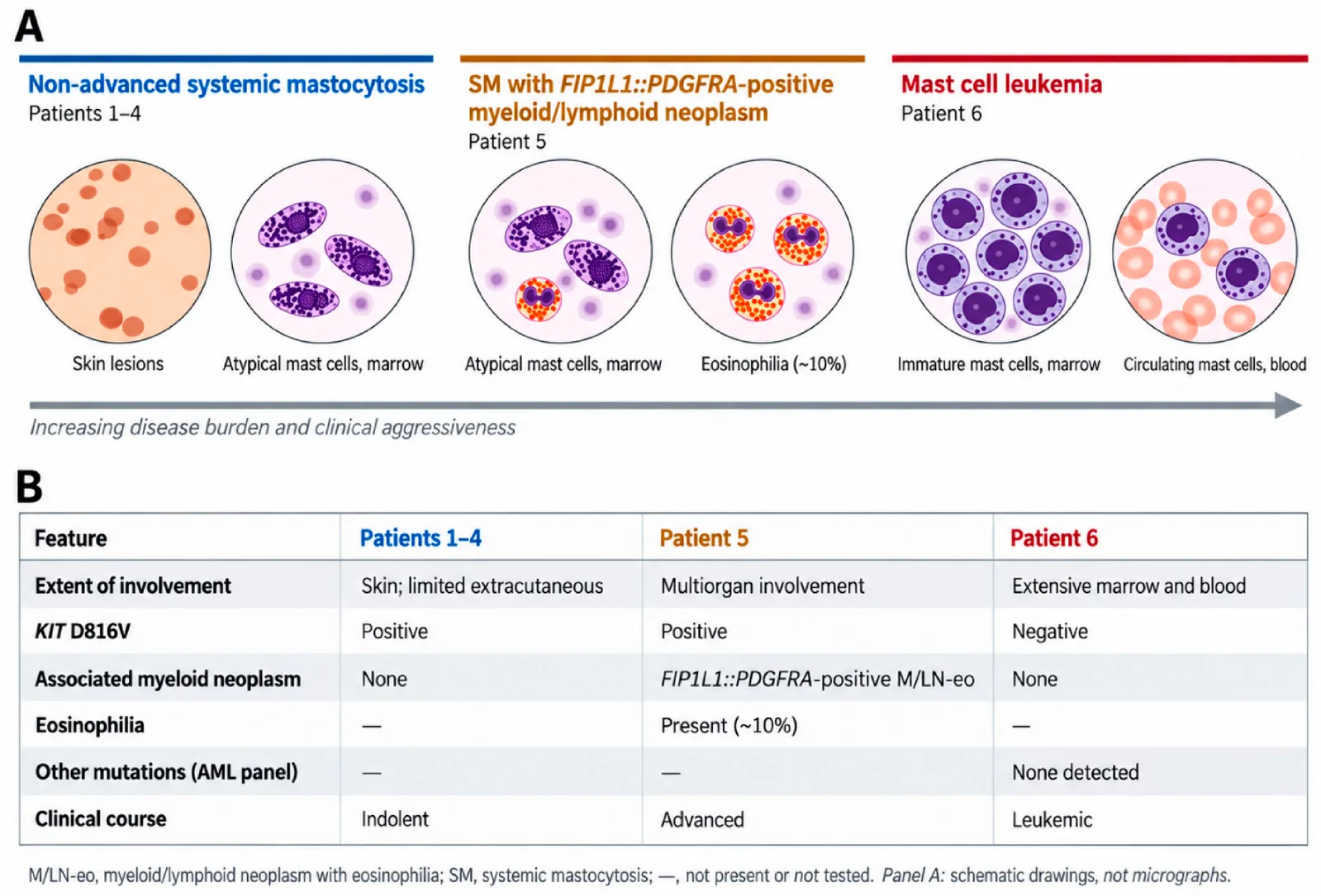
Clinical and biological spectrum of systemic mast-cell disease across the six patients. Patients 1–4 represent non-advanced systemic mastocytosis with predominantly cutaneous and limited extracutaneous involvement; Patient 5 represents a FIP1L1::PDGFRA-positive myeloid/lymphoid neoplasm with eosinophilia and prominent systemic mast-cell involvement; Patient 6 represents mast cell leukemia with extensive marrow and peripheral blood involvement. The colored text distinguishes the three clinical categories: blue indicates non-advanced systemic mastocytosis (Patients 1–4), orange indicates the FIP1L1::PDGFRA-positive myeloid/lymphoid neoplasm with eosinophilia (Patient 5), and red indicates mast cell leukemia (Patient 6).

The six patients demonstrated a broad spectrum of systemic mast-cell disease, ranging from predominantly cutaneous manifestations with limited bone marrow involvement to aggressive hematologic phenotypes associated with extensive organ infiltration and poor outcomes (Figure 12).

Patients 1–4 had non-advanced systemic mastocytosis with variable cutaneous, gastrointestinal, skeletal, and bone marrow involvement. Despite recurrent *KIT* D816V mutations and aberrant mast-cell immunophenotypes, their clinical manifestations and disease burden differed considerably.

Patient 5 had a *FIP1L1::PDGFRA*-positive myeloid/lymphoid neoplasm with eosinophilia and prominent systemic mast-cell involvement, characterized by multiorgan disease and markedly elevated serum tryptase. In contrast, Patient 6 presented with mast cell leukemia, extensive marrow and peripheral blood involvement, and rapid clinical deterioration despite a comparatively modest tryptase elevation.

These findings demonstrate that serum tryptase concentrations alone do not reliably reflect disease aggressiveness. Comprehensive characterization requires integration of clinical manifestations, organ involvement, bone marrow morphology, immunophenotype, and molecular findings.

## 4. Discussion

This single-center real-world series illustrates the broad clinical and biological heterogeneity of systemic mast-cell disease. Across the six patients, bone marrow morphology, immunophenotype, molecular findings, serum tryptase, and organ involvement provided complementary information for disease characterization.

### 4.1. Non-Advanced Systemic Mastocytosis: Position of Patients 1–4, Historically Classified as Smoldering Systemic Mastocytosis According to the 2016 WHO Framework

Smoldering systemic mastocytosis (SSM) is a non-advanced disease subtype characterized by an increased mast-cell burden without organ damage attributable to mast-cell infiltration (C-findings). It represents an intermediate phenotype between indolent and advanced systemic mastocytosis (SM). Although its prognosis is generally more favorable than that of advanced SM, SSM carries a risk of progression. In a European Competence Network on Mastocytosis (ECNM) analysis involving 1993 patients with non-advanced disease, progression occurred in 9.4% of patients with SSM [40].

SSM remains uncommon, accounting for 20 of 460 patients in an Italian multicenter study [41].

In our cohort, Patients 1–4 exhibited bone marrow involvement, *KIT* D816V positivity, and aberrant CD25 and/or CD2 expression, without documented C-findings. However, the retrospective records did not consistently establish at least two B-findings required for SSM classification under contemporary criteria. Therefore, these patients were conservatively categorized as having non-advanced SM to avoid potential overclassification.

Their relatively stable clinical course during the available follow-up supports continued surveillance in the absence of documented progression. These cases also highlight the diagnostic importance of integrating skin histopathology, bone marrow morphology, immunophenotyping, and molecular testing. The importance of timely recognition of cutaneous disease has also been highlighted in other dermatological malignancies. A Romanian retrospective study reported persistent disruptions in melanoma diagnosis and less favorable histopathological prognostic features following the COVID-19 pandemic [42]. Although these findings concern a different disease, they underscore the broader importance of timely dermatological assessment.

The empirical imatinib trial in Patient 1 should be interpreted in its historical therapeutic context. The *KIT* D816V mutation is generally associated with imatinib resistance, consistent with the lack of clinical benefit observed in this patient.

### 4.2. FIP1L1::PDGFRA-Positive Myeloid/Lymphoid Neoplasm with Eosinophilia and Systemic Mast-Cell Disease: Classification and Clinical Relevance

Systemic mastocytosis with an associated hematologic neoplasm (SM-AHN) represents a major category of advanced systemic mastocytosis (SM). In a 2026 European Competence Network on Mastocytosis (ECNM) registry analysis, 467 of 3925 patients (11.9%) had myeloid SM-AHN, with a median overall survival of 36.1 months compared with 340.9 months in patients without an associated hematologic neoplasm [43]. These findings emphasize the prognostic importance of characterizing both disease components.

Patient 5 presented an unusual molecular profile, combining *KIT* D816V-positive systemic mast-cell disease with a *FIP1L1::PDGFRA*-positive myeloid/lymphoid neoplasm with eosinophilia. Historically, similar presentations were described as systemic mast-cell disease with eosinophilia or SM associated with chronic eosinophilic leukemia. Early studies identified *FIP1L1::PDGFRA* in 3 of 5 and 10 of 19 patients, respectively, although these series included overlapping populations [44,45].

Contemporary WHO and International Consensus Classification (ICC) frameworks classify *FIP1L1::PDGFRA*-positive disease primarily among myeloid/lymphoid neoplasms with eosinophilia and defining tyrosine kinase gene fusions, rather than automatically as SM-AHN. This distinction is clinically relevant because *FIP1L1::PDGFRA*-positive neoplasms may exhibit increased mast-cell numbers, elevated serum tryptase, and aberrant mast-cell phenotypes that resemble SM.

The coexistence of *KIT* D816V and *FIP1L1::PDGFRA* raises the possibility of distinct clonal processes, although their relationship could not be established in our patient. A previously reported case described *KIT* D816V-positive SM followed by a *FIP1L1::PDGFRA*-positive myeloid neoplasm that responded to imatinib [46]. Together, these observations illustrate the diagnostic complexity of overlapping molecular abnormalities.

This distinction also has therapeutic implications. Midostaurin targets *KIT*-driven mast-cell disease, whereas *FIP1L1::PDGFRA*-positive neoplasms are generally sensitive to imatinib. Patient 5 received midostaurin, with imatinib subsequently considered for the *FIP1L1::PDGFRA*-positive component. Despite initial improvement, the patient developed bronchopneumonia and cardiorespiratory decompensation following orthopedic surgery, ultimately resulting in death. The terminal deterioration was likely multifactorial and cannot be attributed solely to mast-cell infiltration.

### 4.3. Mast Cell Leukemia: Rarity, Prognosis, and the Diagnostic Dilemma with Acute Basophilic Leukemia

Mast cell leukemia (MCL) is a rare and aggressive form of systemic mastocytosis, accounting for approximately 1% or fewer of cases in historical series. A 2013 review identified 51 cases, while a subsequent European Competence Network on Mastocytosis (ECNM) registry study included 92 patients [47,48]. In the latter cohort, median overall survival was 1.6 years, decreasing to approximately 0.4 years in patients with ≥10% circulating mast cells. *KIT* D816V-negative disease was also associated with poorer outcomes. Patient 6 exhibited several adverse features, including extensive peripheral blood involvement, *KIT* D816V negativity, progressive organ dysfunction, and death approximately five months after diagnosis [49].

Although outcomes have improved in selected patients receiving targeted therapies, prognosis remains poor. In the pivotal midostaurin study, median overall survival in the MCL subgroup was approximately 9.4 months [25,48]. Patient 6 received decitabine followed by decitabine combined with imatinib, achieving only transient hematologic and clinical improvement. Although imatinib may be effective against certain sensitive *KIT* alterations, *KIT* D816V negativity alone does not predict treatment response, highlighting the importance of comprehensive molecular testing [48,49].

The principal diagnostic challenge was distinguishing MCL from acute basophilic leukemia (ABL). Both conditions may exhibit immature cells with basophilic cytoplasm and metachromatic granules, limiting the discriminatory value of toluidine blue staining. ABL typically shows a basophilic/myeloid immunophenotype, frequently expressing CD123, CD203c, and CD11b, whereas strong CD117 and mast-cell tryptase expression favor mast-cell differentiation. Nevertheless, no single marker is sufficiently specific to establish the diagnosis [50].

In Patient 6, flow cytometry identified two immature cell populations with features suggesting different stages of mast-cell-lineage maturation. CD117 positivity, CD203c expression, extensive marrow infiltration, and the overall clinicopathological findings supported MCL despite *KIT* D816V negativity and absent CD2 or predominantly weak/variable CD25 expression. These findings emphasize that the absence of conventional aberrant mast-cell markers does not exclude immature MCL [48,51].

Accurate differentiation between MCL and ABL therefore requires integrated morphological, cytochemical, immunophenotypic, and molecular assessment, including exclusion of *BCR::ABL1*-positive blast phase and other genetically defined acute myeloid leukemias. The principal distinguishing features are summarized in Table 4.

### 4.4. Clinical Implications and What This Series Adds

This series highlights three clinically relevant findings. First, non-advanced systemic mastocytosis (SM) may remain stable despite documented bone marrow clonality, emphasizing the importance of recognizing persistent cutaneous manifestations and ensuring appropriate systemic evaluation. Second, eosinophilia in patients with systemic mast-cell disease warrants molecular investigation, particularly for *FIP1L1::PDGFRA*, which identifies a therapeutically actionable myeloid/lymphoid neoplasm and has important classification implications. Third, mast cell leukemia (MCL) may lack *KIT* D816V and mimic acute basophilic leukemia, requiring integrated morphological, immunophenotypic, and molecular assessment.

**Figure 13:**
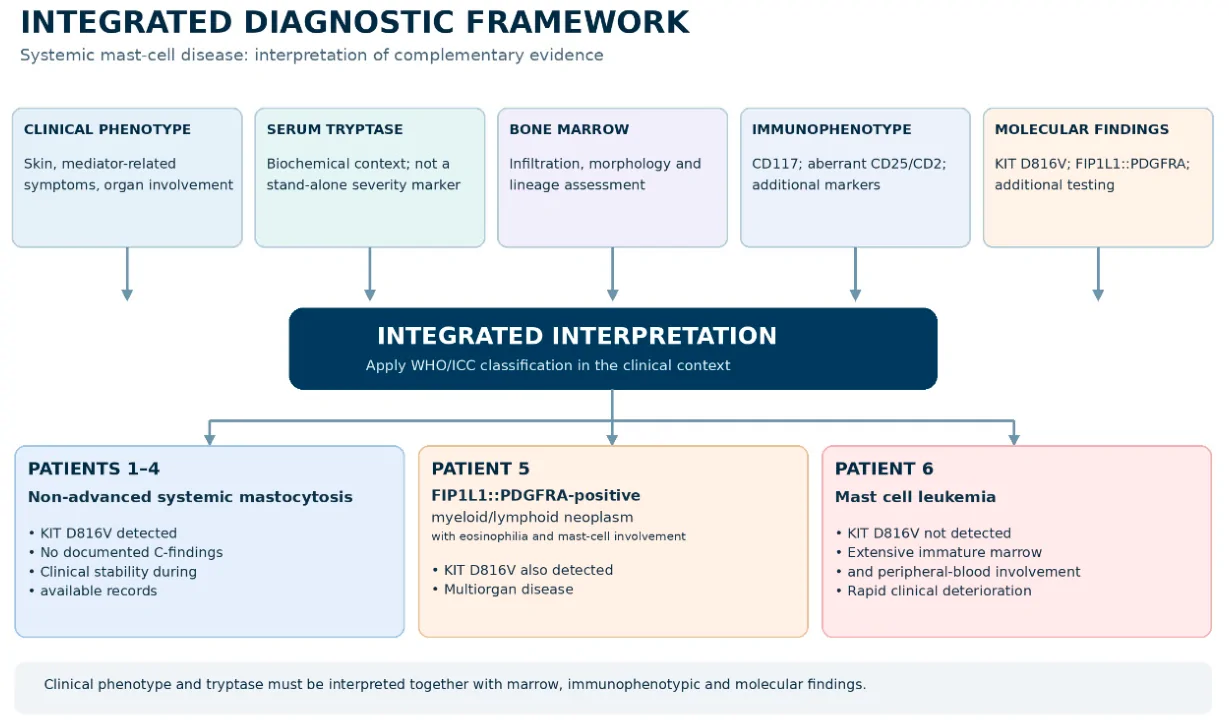
Revised integrated diagnostic framework for systemic mast-cell disease. Clinical phenotype, serum tryptase, bone marrow morphology, immunophenotype, and molecular findings are interpreted together according to contemporary classification frameworks. The diagram illustrates the diagnostic distinction between non-advanced systemic mastocytosis (Patients 1–4), a FIP1L1::PDGFRA-positive myeloid/lymphoid neoplasm with eosinophilia and prominent mast-cell involvement (Patient 5), and mast cell leukemia (Patient 6). The differently colored boxes distinguish the diagnostic components and disease categories. The upper boxes represent the clinical, biochemical, morphological, immunophenotypic, and molecular assessments, while the dark blue box indicates their integrated interpretation. The lower light blue, light orange, and light pink boxes represent non-advanced systemic mastocytosis (Patients 1–4), the FIP1L1::PDGFRA-positive myeloid/lymphoid neoplasm with eosinophilia (Patient 5), and mast cell leukemia (Patient 6), respectively.

A notable observation was the discordance between serum tryptase concentrations and disease aggressiveness. Patient 5 had the highest tryptase concentration, whereas Patient 6 exhibited a substantially lower value despite extensive leukemic involvement. This finding may reflect the immature mast-cell phenotype in MCL, as poorly differentiated neoplastic cells may produce or release less tryptase. Therefore, serum tryptase should not be considered an isolated surrogate for mast-cell burden or disease severity. The integrated diagnostic approach is summarized in Figure 13.

Next-generation sequencing (NGS) provides additional prognostic information, particularly in advanced SM. Mutations involving *SRSF2*, *ASXL1*, and *RUNX1* are associated with adverse outcomes, while alterations in *TET2*, *JAK2*, and other genes may further characterize clonal complexity. However, comprehensive NGS profiling was not routinely available in this retrospective cohort, limiting molecular risk stratification and the assessment of additional prognostic abnormalities.

### 4.5. Strengths and Limitations

The main strength of this study is the comprehensive characterization of a broad spectrum of systemic mast-cell disease within a single center. By integrating clinical findings, histopathology, bone marrow morphology, immunophenotyping, serum tryptase, and molecular testing, this series highlights important diagnostic challenges. The inclusion of a *FIP1L1::PDGFRA*-positive myeloid/lymphoid neoplasm with eosinophilia and prominent systemic mast-cell involvement, alongside a *KIT* D816V-negative mast cell leukemia mimicking acute basophilic leukemia, provides additional descriptive value.

Several limitations should be acknowledged. The small retrospective cohort, comprising six patients diagnosed between 2018 and 2022, limits generalizability and precludes meaningful statistical comparisons. Diagnostic investigations were not fully standardized, while comprehensive next-generation sequencing (NGS) and quantitative *KIT* D816V variant allele frequency (VAF) measurements were not consistently available. Incomplete clinical and molecular data also limited uniform retrospective application of prognostic models, particularly the Mutation-Adjusted Risk Score (MARS).

Clinical outcomes require cautious interpretation. Patients with non-advanced systemic mastocytosis remained under follow-up without documented progression, whereas both patients with advanced hematologic disease died. However, these outcomes cannot be directly compared because of the small sample size, heterogeneous disease phenotypes, and variable follow-up. Moreover, the fatal outcome in Patient 5 followed infectious and cardiorespiratory complications, whereas Patient 6 experienced progressive leukemic disease with recurrent cardiopulmonary deterioration.

Finally, historical classifications of eosinophilic neoplasms with *FIP1L1::PDGFRA* may not correspond directly to contemporary WHO and International Consensus Classification (ICC) categories. Accordingly, comparisons with previous studies should consider molecular findings and diagnostic criteria rather than relying solely on historical terminology.

## 5. Conclusions

This single-center series illustrates the marked clinical and biological heterogeneity of systemic mastocytosis, ranging from non-advanced disease to aggressive hematologic phenotypes. Differences in organ involvement, bone marrow morphology, immunophenotype, molecular findings, and serum tryptase concentrations emphasize the limitations of relying on individual diagnostic parameters.

The identification of a *FIP1L1::PDGFRA*-positive myeloid/lymphoid neoplasm with eosinophilia and prominent systemic mast-cell involvement highlights the importance of molecular testing for accurate classification and therapeutic decision-making. Conversely, the rapidly progressive course of *KIT* D816V-negative mast cell leukemia demonstrates the diagnostic challenges posed by immature mast-cell proliferations, particularly when distinguishing them from acute basophilic leukemia.

Despite the limitations of a small retrospective cohort, these findings support an integrated multidisciplinary approach combining clinical assessment, histopathology, immunophenotyping, serum biomarkers, and molecular testing to improve disease characterization and guide individualized management.

## Figures and Tables

**Table 1:** Demographic, clinical, and diagnostic characteristics of the six patients.

Patient	Sex	Age	Main Presentation	Serum tryptase (μg/L)	Molecular Status	Classification
P1	F	54	Telangiectatic skin lesions, pruritus, GI symptoms	105	KIT D816V+	Non-advanced SM
P2	M	27	Thoracoabdominal telangiectatic and maculopapular lesions	15.8	KIT D816V+ (weakly positive)	Non-advanced SM
P3	M	37	Pink–violaceous maculopapular rash	30.3	KIT D816V+	Non-advanced SM
P4	M	16	Disseminated maculopapular and telangiectatic lesions	23.1	KIT D816V+	Non-advanced SM
P5	M	79	Pruritic skin lesions, constitutional symptoms, weight loss	348	*KIT D816V+; FIP1L1::PDGFRA+*	FIP1L1::PDGFRA-positive myeloid/lymphoid neoplasm with eosinophilia and prominent systemic mast-cell involvement
P6	F	86	Anasarca, dyspnea, cardiorespiratory failure	28.5	*KIT D816V−*	MCL

Abbreviations: F, female; M, male; GI, gastrointestinal; SM, systemic mastocytosis; MCL, mast cell leukemia.

**Table 2:** Bone marrow morphology, immunophenotype, and molecular findings in the six patients.

Patient	Bone Marrow Morphology	Immunophenotype	Molecular Findings
P1	Approximately 5–6% atypical mast cells overall; up to 42% in marrow particles; nodular mast-cell infiltrates	CD117+, CD25+	*KIT* D816V+
P2	Approximately 3% atypical spindle-shaped mast cells; 8–15% in marrow particles; focal aggregate up to 50%	CD117++, CD25+, CD2+	*KIT* D816V+ (weakly positive)
P3	Predominantly type I atypical mast cells with fewer type II forms; approximately 2–5% in myeloid tissue and up to 15% in marrow particles	CD117++, CD203c+, CD25+, CD2+; variable/weak HLA-DR, CD34, CD123	*KIT* D816V+
P4	Type I and type II mast cells; approximately 1–3% in myeloid tissue and 13% in marrow particles; focal medullary infiltration	CD117++, CD203c+, CD25+, CD2+; flow cytometry suggestive of SM	*KIT* D816V+
P5	Mast-cell and granulocytic hyperplasia; 2–16% mast cells, >25% in some marrow particles; eosinophils approximately 10%	CD117+, CD45+, CD203c+, CD25+, CD2+	*KIT* D816V+; FIP1L1::PDGFRA+
P6	Hypercellular marrow with approximately 65% blast-like cells; toluidine blue positive in approximately 30% of marrow cells	Two immature mast-cell-lineage subpopulations; CD34+, CD117+, CD13+ with variable HLA-DR, CD123, CD203c, CD25, CD56, CD9, CD22, and CD35	*KIT* D816V−; *FLT3*, *BCR::ABL1*, *NPM1*, *RUNX1::RUNX1T1* (AML1-ETO), and *CBFB* rearrangements negative

Abbreviations: BM, bone marrow; SM, systemic mastocytosis; MCL, mast cell leukemia; AML, acute myeloid leukemia; HLA-DR, human leukocyte antigen-DR.

**Table 3:** Symptom-control treatment, disease-modifying therapy, and clinical outcomes.

Patient	Symptom-Control Therapy	Disease-Modifying Therapy	Documented Response	Clinical Outcome
P1	Sodium cromoglycate, famotidine, trigger avoidance	Imatinib 100 mg/day for ~3 months	No meaningful clinical benefit; imatinib discontinued	Ongoing follow-up and surveillance
P2	Sodium cromoglycate, famotidine, trigger avoidance	None	Clinically stable	Ongoing follow-up; orthopedic surgery without mast-cell-related complications
P3	Symptom-directed anti-mediator therapy	None	Clinically stable	Ongoing follow-up
P4	Sodium cromoglycate, famotidine, trigger avoidance	None	Clinically stable	Ongoing follow-up; allergy evaluation before vaccination
P5	Sodium cromoglycate, famotidine, sucralfate, supportive therapy	Midostaurin; imatinib considered but not documented as administered for the *FIP1L1::PDGFRA*-positive component	Initial clinical improvement, followed by infectious and cardiorespiratory deterioration	Hip fracture surgery; subsequent bronchopneumonia, intensive care unit (ICU) admission, cardiorespiratory failure, and death
P6	Supportive therapy	Decitabine, followed by decitabine + imatinib for 4 cycles	Transient reduction in leukocytes and circulating blasts, without durable response	Progressive disease and death approximately 5 months after diagnosis

**Table 4:** Practical differential diagnosis of mast cell leukemia and acute basophilic leukemia.

Feature	Mast Cell Leukemia (MCL)	Acute Basophilic Leukemia (ABL)
Lineage/context	Leukemic mast-cell neoplasm; ≥20% mast cells in bone marrow aspirate	Rare basophilic-lineage leukemia; SM criteria not required
Morphology	Immature or atypical mast cells, occasionally hypogranular or blast-like	Basophilic blasts with coarse granules; morphological overlap possible
Tryptase	Usually strong/positive	Usually absent or weak; variable expression possible
CD117 (KIT)	Usually strongly positive	Usually negative or weak
CD123	Variable or weak	Typically positive/bright
CD203c	Variable; may be positive	Frequently positive
CD11b	Usually negative	Frequently positive
CD2/CD25	May be aberrantly expressed; potentially absent in immature MCL	Usually negative; occasional CD25 expression
MPO	Negative	Usually negative
Toluidine blue	Positive; not lineage-specific	Positive; not lineage-specific
*KIT* mutation	D816V common in SM but not obligatory in MCL; other variants possible	*KIT* D816V generally absent
Molecular evaluation	Comprehensive KIT assessment and exclusion of other myeloid neoplasms	Exclusion of BCR::ABL1-positive blast phase and AML-defining genetic abnormalities
Integrated interpretation	CD117/tryptase expression, mast-cell-lineage phenotype, and clinicopathological context	Basophilic morphology, CD123/CD203c/CD11b expression, and usually weak/absent CD117

Abbreviations: MCL, mast cell leukemia; ABL, acute basophilic leukemia; AML, acute myeloid leukemia; MPO, myeloperoxidase; SM, systemic mastocytosis.

## Data Availability

The data supporting the findings of this study are available from the corresponding author upon reasonable request. The data are not publicly available because of privacy considerations related to the detailed clinical information of patients with a rare disease.
